# Cationic-nanogel nasal vaccine containing the ectodomain of RSV-small hydrophobic protein induces protective immunity in rodents

**DOI:** 10.1038/s41541-023-00700-3

**Published:** 2023-07-24

**Authors:** Shingo Umemoto, Rika Nakahashi-Ouchida, Yoshikazu Yuki, Shiho Kurokawa, Tomonori Machita, Yohei Uchida, Hiromi Mori, Tomoyuki Yamanoue, Takehiko Shibata, Shin-ichi Sawada, Kazuya Ishige, Takashi Hirano, Kohtaro Fujihashi, Kazunari Akiyoshi, Yosuke Kurashima, Daisuke Tokuhara, Peter B Ernst, Masashi Suzuki, Hiroshi Kiyono

**Affiliations:** 1 Division of Mucosal Immunology, IMSUT Distinguished Professor Unit, The Institute of Medical Science, The University of Tokyo, Tokyo, Japan; 2 Department of Otorhinolaryngology & Head and Neck Surgery, Faculty of Medicine, Oita University, Oita, Japan; 3 Chiba University-University of California San Diego Center for Mucosal Immunology, Allergy and Vaccine (CU-UCSD cMAV), Department of Medicine, School of Medicine, San Diego, CA USA; 4 Division of Mucosal Vaccines, International Research and Development Center for Mucosal Vaccines, The Institute of Medical Science, The University of Tokyo, Tokyo, Japan; 5 Department of Human Mucosal Vaccinology, Chiba University Hospital, Chiba, Japan; 6 Chiba University Synergy Institute for Futuristic Mucosal Vaccine Research and Development, Chiba University, Chiba, Japan; 7 HanaVax Inc, Tokyo, Japan; 8 Department of Microbiology, Tokyo Medical University, Tokyo, Japan; 9 Department of Immunology, National Institute of Infectious Diseases, Tokyo, Japan; 10 Department of Polymer Chemistry, Graduate School of Engineering, Kyoto University, Kyoto, Japan; 11 Biochemicals Division, Yamasa Corporation, Chiba, Japan; 12 Division of Mucosal Vaccines, International Vaccine Design Center, The Institute of Medical Science, The University of Tokyo, Tokyo, Japan; 13 Department of Pediatric Dentistry, The University of Alabama at Birmingham, Birmingham, AL USA; 14 Institute for Advanced Academic Research, Chiba University, Chiba, Japan; 15 Department of Innovative Medicine, Graduate School of Medicine, Chiba University, Chiba, Japan; 16 Department of Pediatrics, Wakayama Medical University, Wakayama, Japan; 17 Division of Comparative Pathology and Medicine, Department of Pathology, University of California, San Diego, CA USA; 18 Center for Veterinary Sciences and Comparative Medicine, University of California, San Diego, CA USA; 19 Future Medicine Education and Research Organization, Chiba University, Chiba, Japan; 20 Mucosal Immunology and Allergy Therapeutics, Institute for Global Prominent Research, Chiba University, Chiba, Japan

**Keywords:** Protein vaccines, Protein vaccines

## Abstract

Respiratory syncytial virus (RSV) is a leading cause of upper and lower respiratory tract infection, especially in children and the elderly. Various vaccines containing the major transmembrane surface proteins of RSV (proteins F and G) have been tested; however, they have either afforded inadequate protection or are associated with the risk of vaccine-enhanced disease (VED). Recently, F protein-based maternal immunization and vaccines for elderly patients have shown promising results in phase III clinical trials, however, these vaccines have been administered by injection. Here, we examined the potential of using the ectodomain of small hydrophobic protein (SHe), also an RSV transmembrane surface protein, as a nasal vaccine antigen. A vaccine was formulated using our previously developed cationic cholesteryl-group-bearing pullulan nanogel as the delivery system, and SHe was linked in triplicate to pneumococcal surface protein A as a carrier protein. Nasal immunization of mice and cotton rats induced both SHe-specific serum IgG and mucosal IgA antibodies, preventing viral invasion in both the upper and lower respiratory tracts without inducing VED. Moreover, nasal immunization induced greater protective immunity against RSV in the upper respiratory tract than did systemic immunization, suggesting a critical role for mucosal RSV-specific IgA responses in viral elimination at the airway epithelium. Thus, our nasal vaccine induced effective protection against RSV infection in the airway mucosa and is therefore a promising vaccine candidate for further development.

## Introduction

Respiratory syncytial virus (RSV) is a leading cause of upper and lower respiratory tract infection, especially in children younger than five^1^ and the elderly^2^. RSV bronchiolitis and pneumonia in infancy promote the development of asthma and allergy^3^. High-risk children (e.g., those with congenital heart disease or immune deficiency) often develop severe, sometimes lethal, complications due to RSV infection^4,5^, and the same applies to high-risk adults^6^, Currently, the only available therapeutic agent against RSV infection is a humanized IgG1 monoclonal antibody targeting the fusion (F) glycoprotein of RSV, and it is only approved for use in high-risk children^7,8^. It is also an expensive, passive immunization that requires repeated monthly intramuscular injections, placing economic, physical, and psychological burdens on patients, their families, and health care systems^9,10^. Thus, an active immunization strategy, a prophylactic RSV vaccine, is urgently needed.

In the 1960s, clinical trials of a formalin-inactivated (FI)-RSV vaccine that induced neutralizing antibodies were conducted; however, the vaccine failed to provide sufficient protection, leaving immunized infants vulnerable to severe, sometimes lethal, clinical complications after natural RSV infection, a condition known as vaccine-enhanced disease (VED)^11,12^. Since then, many experimental vaccines have been investigated using subunits of two of the major RSV surface glycoproteins, protein F^13,14^ and the attachment glycoprotein (protein G)^13,15^, or live-attenuated RSV^16–18^ as candidate antigens. Recently, several promising vaccine candidates have been developed and are currently under clinical investigation^19^, with particularly encouraging results coming from the phase III trials of F protein-based vaccines^20–22^. However, the injectable vaccine is generally less effective for the induction of protective immunity at the RSV invasion site of airway mucosal surface, while the nasal vaccine has been shown to induce both mucosal and systemic immune responses against respiratory pathogens^23–27^.

In addition to the major surface glycoproteins F and G, the RSV envelope contains another viral transmembrane surface glycoprotein: small hydrophobic (SH) protein^28,29^. Compared with antibodies to proteins F and G^29^, antibodies to SH protein show less neutralizing activity^30^ most likely because the SH protein is not involved in the initiation of virus infection and it has low immunogenicity^31^. However, RSV replication is reduced without the induction of VED via an antibody-dependent cellular cytotoxicity (ADCC) mechanism involving SH-specific IgG antibodies^30,32^. Because of this lower induction of neutralizing activity by SH antigen^31,33,34^, SH-based antigens are generally considered less-than-attractive subunit antigens for RSV vaccine development, despite their advantage of not inducing VED.

Previously, we developed a nanometer-sized hydrogel, a “nanogel”, consisting of a cationic cholesteryl-group-bearing pullulan (cCHP). We found that our cCHP-nanogel is a safe and effective nasal vaccine delivery system that can be used to stimulate the immune system of the nasal mucosa by using a variety of subunit antigens^23–26,35–37^. Because of its cationic property, cCHP-nanogel shows persistent attachment to the surfaces of the negatively charged nasal mucosa, leading to prolonged release of antigen to the antigen-sampling and—presenting systems of the nasal mucosa^38^.

Because RSV initially infects the respiratory mucosa, it is logical to confer protective immunity not only in the systemic compartment but also at the respiratory mucosa (e.g., nasal cavity and lung). We therefore decided to take advantage of the safety of SH-based antigens by combining one with our nanogel-based vaccine delivery vehicle and produced a cCHP-based nasal subunit vaccine containing the ectodomain of SH protein (SHe) as the antigen. We then examined whether cCHP-based SHe nasal vaccine conferred effective protection in the airway mucosa of mice and cotton rats without inducing VED and whether this formulation could be considered a potent vaccine candidate for further clinical development.

## Results

### Design and synthesis of the SHe antigen

**Fig. 1:**
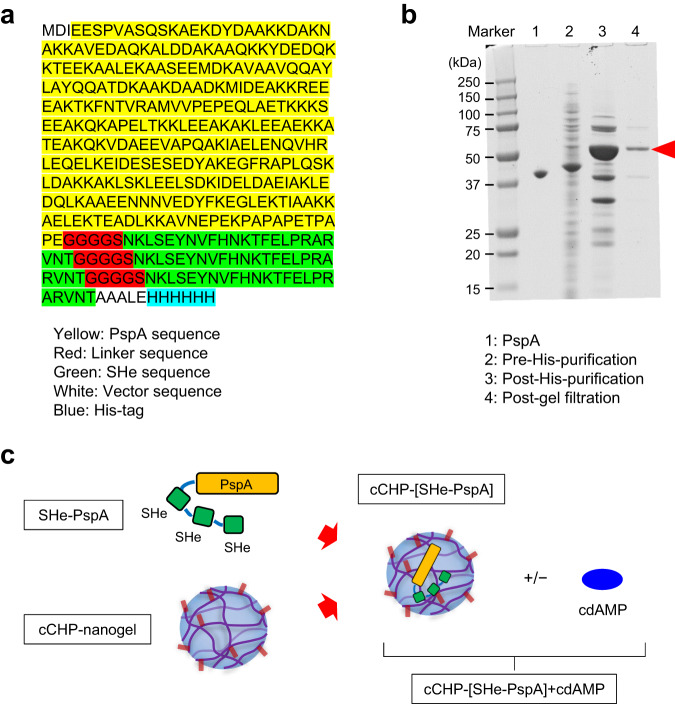
Production of cCHP-[SHe-PspA]. **a** Mass spectrometry analysis revealed that the *Escherichia coli*-derived recombinant protein contained the complete sequences of PspA (yellow), triplicate linkers (red), triplicate ectodomains of small hydrophobic protein (green), vector (white), and his-tag (blue). **b** A chimeric, His-tagged, PspA-conjugated antigen was produced in *Escherichia coli*. The protein was purified by metal chelate chromatography followed by gel filtration. Boiled samples at the stages of purification were separated by SDS-PAGE. Protein bands were visualized by Coomassie brilliant blue staining. After sample purification, we detected a 44-kDa protein corresponding to the SHe-PspA protein (red arrowhead). **c** Schematic of the vaccine design. Three repeated SHe sequences were conjugated with PspA to enhance the immunogenicity of the antigen.

For use as the antigen in our nasal vaccine, we used the peptide sequence of the ectodomain of the SH protein of RSV subtype A (Fig. 1a). The antigen was constructed by using pneumococcal surface protein A (PspA) of *Streptococcus pneumoniae* as the carrier protein^23,25,35–37^. Because the native SH protein has low immunogenicity^31,33,34^, we enhanced the immunogenicity of the vaccine antigen by linking three of the SHe peptides in sequence to the carrier protein. The complete vaccine antigen was obtained by using an *Escherichia coli* expression system and purified by His-tag purification (Fig. 1b), and its amino acid sequence was confirmed by mass spectrometry analysis. Then, the SHe-PspA antigen was formulated with cCHP-nanogel in a 1:1 antigen:nanogel molar ratio, in the presence or absence of cyclic-di-AMP (cdAMP) as an adjuvant, and used in the subsequent nasal immunization studies (Fig. 1c).

### Nasal immunization with cCHP-[SHe-PspA] induces SHe-specific immune responses in the systemic compartment and on the mucosal surface

**Fig. 2:**
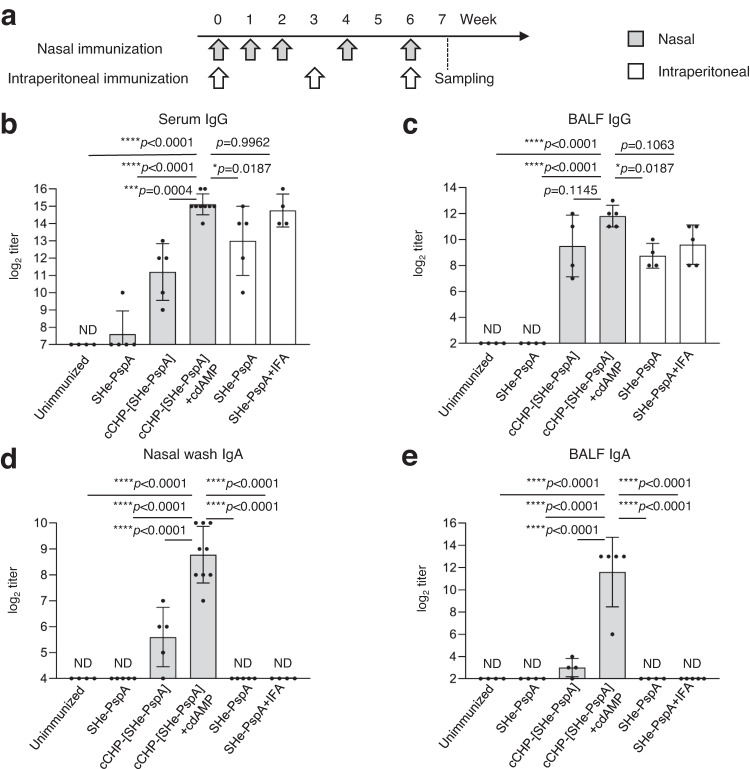
Nasal immunization with cCHP-[SHe-PspA] induces SHe-specific antibody responses in mice. **a** Immunization schedule. Wild-type (WT) female BALB/c mice were nasally immunized for three times at weekly intervals and then boosted two times at 2-week intervals. Other WT BALB/c mice were intraperitoneally immunized every 3 weeks a total of three times. Control mice (unimmunized) received phosphate-buffered saline administered intranasally. **b**–**e** SHe-specific antibody titers one week after the final immunization in serum (**b**), nasal wash (**d**), and bronchoalveolar lavage fluid (BALF) (**c**, **e**). Data are representative of five or more independent experiments, and each group consisted of seven or more mice. Each black circle represents an individual mouse. ND, not detected in undiluted samples. One-way analysis of variance (ANOVA) with Tukey’s multiple comparison test was used for statistical analysis. NS, not significant; *p < 0.05; ***p < 0.001; ****p < 0.0001. Values are presented as means ± 1 SD.

To investigate whether nasal vaccination with our cCHP-based SHe-PspA antigen (cCHP-[SHe-PspA]) induces SHe-specific antibody responses, we determined SHe-specific antibody titers in serum, nasal wash, and bronchoalveolar lavage fluid (BALF) collected from mice nasally immunized (NI) with phosphate-buffered saline (PBS, unimmunized), SHe-PspA alone, or cCHP-[SHe-PspA] with or without cdAMP (Fig. 2a and Table 1). In addition, we determined the antibody titers in samples collected from mice intraperitoneally immunized (iPI) with SHe-PspA plus incomplete Freund’s adjuvant (IFA) or SHe-PspA alone.

**Table 1:** Treatment groups used in mouse experiments.

Mouse treatment groups	SHe-PspA	Adjuvant	Immunization weeks
	Per immunization		
*Unimmunized*			
PBS nasal administration	–	–	–
*Nasal immunization*			
SHe-PspA	10 µg	–	0, 1, 2, 4, 6
cCHP-[SHe-PspA]	10 µg	–	0, 1, 2, 4, 6
cCHP-[SHe-PspA] + cdAMP	10 µg	cdAMP 10 µg	0, 1, 2, 4, 6
*Intraperitoneal immunization*			
SHe-PspA	10 µg	–	0, 3, 6
SHe-PspA + IFA	10 µg	IFA 100 µL	0, 3, 6

SHe-specific IgG titers were examined in serum and BALF (Fig. 2b, c). In mice NI with cCHP-[SHe-PspA]+cdAMP, SHe-specific serum and BALF IgG titers were significantly higher than those in mice NI with cCHP-[SHe-PspA] or SHe-PspA alone. Moreover, the serum and BALF titers of mice NI with cCHP-[SHe-PspA]+cdAMP were as high as those of mice iPI with SHe-PspA+IFA. SHe-specific IgA titers were examined in nasal wash and BALF (Fig. 2d, e). Mice NI with cCHP-[SHe-PspA] with or without cdAMP showed high SHe-specific IgA titers in both nasal wash and BALF. The mice that received the adjuvant-containing formulation showed a significantly higher IgA titer than any of the other mice, irrespective of administration route. In fact, the mice in the other groups showed very little to no induction of IgA production. Thus, NI with cCHP-[SHe-PspA] with or without cdAMP induced high titers of SHe-specific serum IgG and mucosal IgA antibodies. In addition, when T cell responses were examined, granzyme B- or interferon (IFN)-γ-producing antigen-specific T cells were induced in lungs, cervical lymph nodes, and spleens of mice immunized with cCHP-[SHe-PspA] based nasal vaccine (Supplemental Fig. 1).

### Nasal immunization with cCHP-[SHe-PspA] induces protective immunity against RSV infection

To evaluate whether immunization with cCHP-[SHe-PspA] provides protective immunity against infection in the upper and lower respiratory tracts, mice were NI with PBS, cCHP-[SHe-PspA], or cCHP-[SHe-PspA]+cdAMP and then challenged with RSV. In addition, mice iPI with SHe-PspA+IFA were also challenged. One week after the final immunization, mice were challenged with the RSV A2 strain intratracheally with 1 × 10^6^ PFU^39^ to evaluate vaccine efficacy in the lungs or intranasally with 1 × 10^5^ PFU^40^ to evaluate vaccine efficacy in the nasal compartments. Four days after the viral challenge, the viral load in the lungs or nasal compartments of the mice was examined by immunoplaque assay (Supplemental Fig. 2a). In addition, the level of RSV-F and -G mRNA was quantified by RT-PCR and compared among these different immunized groups.

**Fig. 3:**
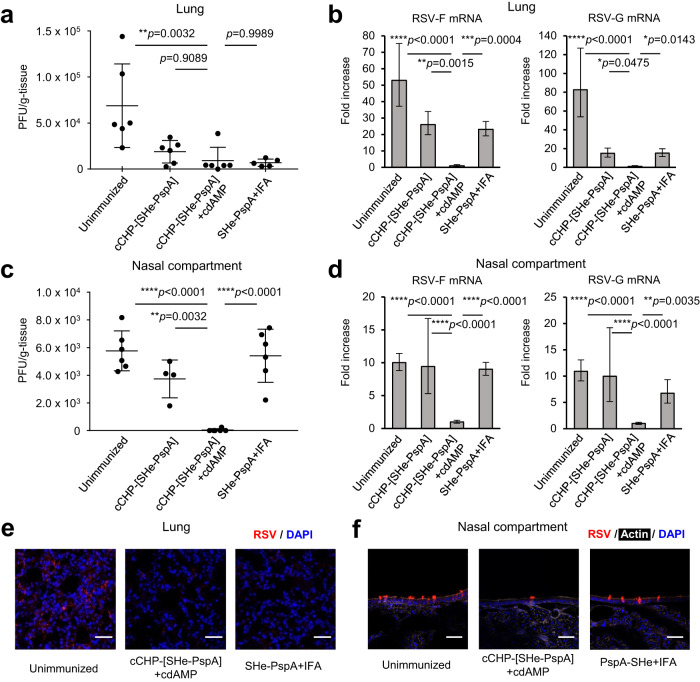
cCHP-[SHe-PspA] induces protective immunity against RSV infection in mice. **a**–**d** One week after the final immunization, mice were challenged with the RSV A2 strain, intratracheally or intranasally, to evaluate vaccine efficacy in the lungs (**a**, **b**) and nasal compartments (**c**, **d**), respectively. Viral clearance was determined by immunoplaque assay (**a**, **c**) or real-time RT-PCR (**b**, **d**). Data are representative of three or more independent experiments, and each group consisted of four or more mice. One-way analysis of variance (ANOVA) with Tukey’s multiple comparison test was used for statistical analysis. NS, not significant; *p < 0.05; **p < 0.01; ***p < 0.001; ****p < 0.0001. Values are presented as means1 SD. **e**, **f** Localization of RSV after infection in lungs (**e**) and nasal compartments (**f**), as detected by immunofluorescence assay. Scale bars: 50 µm. Tissue sections were prepared and stained with anti-RSV (Cy3 [red]), DAPI (blue), and phalloidin (iFluro647 [white]). Red spots indicate RSV invasion. Data are representative of three independent experiments, and each group consisted of two or three mice.

Because the lower respiratory tract is a major site of tissue damage caused by severe RSV infection^5^, we first examined the viral load in the lungs of mice NI or iPI with the various vaccine formulations. Immunoplaque analysis revealed that mice NI with cCHP-[SHe-PspA]+cdAMP had significantly lower viral titers compared with those of the unimmunized mice, and comparable to those of the other immunized mice (Fig. 3a). The viral load in the unimmunized mice was 6.9 × 10^5^ ± 4.5 × 10^5^. In contrast, the cCHP-[SHe-PspA] NI, cCHP-[SHe-PspA]+cdAMP NI, or [SHe-PspA]+IFA iPI immunized mice showed reductions of viral load (1.9 × 10^5^ ± 1.2 × 10^5^, 9.1 × 10^4^ ± 1.5 × 10^4^, and 6.9 × 10^4^ ± 4.0 × 10^4^ PFU/g, respectively). RT-PCR analysis confirmed the immunoplaque result (Fig. 3b). In the nasal compartment, both immunoplaque and RT-PCR analysis showed that mice NI with cCHP-[SHe-PspA]+cdAMP had significantly lower viral titers compared with those of the other mice, (Fig. 3c, d). The viral load in the unimmunized mice was 5.8 × 10^4^ ± 1.4 × 10^4^. In contrast, the cCHP-[SHe-PspA] NI, cCHP-[SHe-PspA]+cdAMP NI, and [SHe-PspA]+IFA iPI immunized mice showed reductions of viral load (3.7 × 10^4^ ± 1.4 × 10^4^, 4.0 × 10 ± 9.8 × 10, and 5.4 × 10^3^ ± 1.9 × 10^3^ PFU/g, respectively). In fact, the viral load in the nasal compartments of mice NI with cCHP-[SHe-PspA]+cdAMP was almost below the limit of detection (4.0 × 10 ± 9.8 × 10 PFU).

Next, we assessed viral infiltration into lung and nasal compartment tissue by means of fluorescent immunostaining of samples obtained from mice NI with PBS or cCHP-[SHe-PspA] with cdAMP or mice iPI with SHe-PspA+IFA (Fig. 3e, f). At 4 days after viral challenge, mice NI with cCHP-[SHe-PspA]+cdAMP or iPI with SHe-PspA+IFA showed almost no viral infiltration in the lung (Fig. 3e). In the nasal compartments, mice NI with cCHP-[SHe-PspA]+cdAMP showed less viral infiltration compared with that in mice iPI with SHe-PspA+IFA (Fig. 3f).

### cCHP-[SHe-PspA] induces antigen-specific antibodies with inhibitory activity against viral invasion in the upper respiratory tract

So far, our results show that cCHP-[SHe-PspA] effectively induces SHe-specific IgA antibody responses in airway tract secretions (Fig. 2c, e). Next, we used a virus-binding bead assay and fluorescence-activated cell sorting^36^ to examine whether the induced antibodies^41^ directly bind to RSV (Supplemental Fig. 3). RSV A2 was conjugated to strong anion exchange virus-binding beads and incubated with mouse nasal wash samples (70 µL/sample) collected from wild-type (WT) BALB/c mice NI with PBS, cCHP-[SHe-PspA], or cCHP-[SHe-PspA]+cdAMP. In addition, nasal wash samples from polymeric Ig receptor deficient (pIgR^−/−^) BALB/c mice NI with cCHP-[SHe-PspA]+cdAMP were examined to directly address the role of SHe-specific mucosal IgA antibodies.

**Fig. 4:**
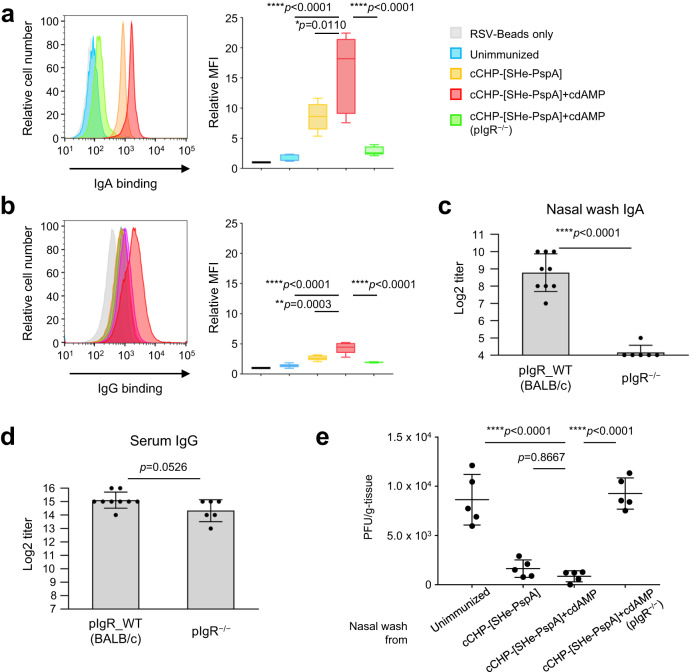
cCHP-[SHe-PspA] inhibits viral invasion in the upper respiratory tract. **a**, **b** Binding on the surface of RSV by nasal wash SHe-specific IgA (**a**) and IgG (**b**) was detected by binding-beads and fluorescence-activated cell sorting analysis. RSV A2 was conjugated with virus-binding beads (RSV-Beads) and incubated with mouse nasal wash. Antibody binding was evaluated by measuring the intensity of fluorescent labeling by flow cytometry, and the mean fluorescent intensity (MFI) of each group was measured and determined as relative MFI with the value for RSV-Beads alone set to 1. Data are representative of three independent experiments, and each group consisted of five or six mice. **c**, **d** Nasal wash SHe-specific IgA (**c**) and serum SHe-specific IgG (**d**) in pIgR WT (BALB/c) and pIgR^−/−^ mice. **e** Immunization-naïve BALB/c mice were challenged intranasally with RSV incubated with nasal wash from immunized mice. Four days later, the viral titer of the nasal compartments was determined by plaque assay. Data are representative of three or more independent experiments, and each group consisted of four or more mice (**a**–**e**). One-way analysis of variance (ANOVA) with Tukey’s multiple comparison test (**a**, **b**, **e**) and two-tailed Student’s *t* test (**c**, **d**) were used for statistical analysis. *p < 0.05; **p < 0.01; ***p < 0.001; ****p < 0.0001. Values are presented as means ± 1 SD.

As expected, nasal washes from the NI WT mice contained significantly higher levels of SHe-specific IgA and IgG antibodies compared with those in WT mice immunized with PBS, which showed very low to undetectable antibody levels as described above (Fig. 2b–e). In addition, antibodies collected from NI mice were found to directly bind to the surface of RSV A2 (Fig. 4a, b). The RSV-binding capacities of antigen-specific IgA and IgG antibodies in nasal washes from NI mice immunized with cCHP-[SHe-PspA]+cdAMP were stronger than those of IgA and IgG from NI mice immunized with cCHP-[SHe-PspA] without cdAMP. In addition, when RSV binding ability was compared between the two isotypes in nasal washes from different NI groups, binding ability of the IgA antibody was greater than that of the IgG antibody (Fig. 4a, b). These findings indicate that nasally induced antigen-specific IgA antibodies in the nasal compartments play a critical role in inhibiting the binding of RSV to the airway mucosal cavity.

To further confirm the inhibitory role of nasal cavity IgA against RSV binding, pIgR^−/−^ BALB/c mice, which lack the ability to produce antigen-specific mucosal secretory IgA^42,43^, were NI with cCHP-[SHe-PspA]+cdAMP and were found to show significantly lower to undetectable levels of SHe-specific IgA in nasal washes compared with those of WT mice (Fig. 4c). In contrast, the same levels of SHe-specific serum IgG were seen in both pIgR^−/−^ and WT BALB/c mice (Fig. 4d). Although nasal washes from NI pIgR^−/−^ mice contained antigen-specific IgG capable of binding to RSV, the antibody’s binding ability was as weak as that of IgG in PBS nasally administered (unimmunized) mice (Fig. 4a, b). These findings support the earlier finding that antigen-specific IgA in the nasal compartments plays a critical role in preventing RSV binding to the upper respiratory mucosa.

To demonstrate the critical role of IgA antibodies in the nasal cavity for the control of upper respiratory tract RSV infection, RSV was pre-incubated with the same nasal wash samples obtained from the BALB/c mice (e.g., WT and pIgR^−/−^) NI with cCHP-[SHe-PspA]+cdAMP. Nasal washes from the mice NI with PBS were used as a control. The pretreated RSV were then used for intranasal challenge of immunization-naïve BALB/c mice (Supplemental Fig. 4). Four days after intranasal challenge, viral titers in the nasal compartments were determined by immunoplaque assay. Compared with the nasal washes from mice NI with PBS, those from WT BALB/c mice NI with cCHP-[SHe-PspA] with or without cdAMP resulted in significantly reduced levels of RSV invasion (Fig. 4e), whereas nasal washes from pIgR^−/−^ mice NI with cCHP-[SHe-PspA]+cdAMP containing antigen-specific IgG, but not IgA, antibodies had no effect on viral invasion (Fig. 4e). The viral load in the mice that received nasal wash from WT BALB/c mice NI with PBS (i.e., unimmunized mice) was 8.6 × 10^4^ ± 2.6 × 10^4^ PFU/g. In contrast, reductions of viral loads were observed in the mice that received nasal washes from WT BALB/c mice NI with cCHP-[SHe-PspA] (1.6 × 10^4^ ± 8.9 × 10^3^ PFU/g) and WT BALB/c mice NI with cCHP-[SHe-PspA]+cdAMP (8.6 × 10^3^ ± 5.6 × 10^3^ PFU/g). It should be noted that the viral load in mice that received nasal washes from secretory IgA-lacking pIgR^−/−^ mice NI with cCHP-[SHe-PspA]+cdAMP were elevated (9.3 × 10^4^ ± 1.6 × 10^4^ PFU/g). These results demonstrate that nasal SHe-specific IgA antibodies play an important role in inhibiting RSV infection at the site of invasion, the airway mucosal surface.

### Lung immune protection induced by cCHP-[SHe-PspA] depends on an Fc receptor-mediated immune mechanism

In addition to the induction of airway mucosal protective immunity (Fig. 3), our results demonstrate that cCHP-[SHe-PspA] induces antigen-specific serum IgG antibody responses equivalent to those induced by systemic immunization (Figs. 2b, d, 3a, b, e). Building on these findings, we used an in vitro plaque reduction assay to examine whether SHe-specific serum IgG and nasal wash IgA from immunized mice are able to directly neutralize RSV; however, neither antibody was found to exert a direct neutralizing effect against RSV (Supplemental Fig. 5), as previously reported^30,31^. We therefore next analyzed the cellular and molecular mechanisms of the systemic protection elicited by cCHP-[SHe-PspA] against RSV.

**Fig. 5:**
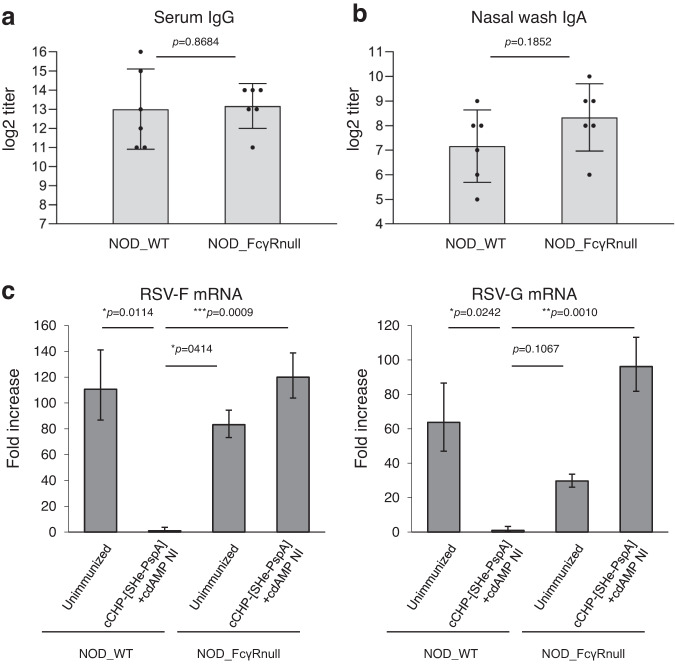
SHe-specific IgG protects against RSV via an Fc receptor-mediated immune mechanism. One week after the final immunization, NOD WT mice and NOD FcγR-null mice were intratracheally challenged with the RSV A2 strain. **a**, **b** Serum SHe-specific IgG and nasal-wash SHe-specific IgA in NOD WT and NOD FcγR-null mice. Data are representative of three independent experiments, and each group consisted of six mice. **c** Viral clearance from the lungs, as determined by detecting RSV-F and -G mRNA using real-time RT-PCR. Data are representative of three independent experiments, and each group consisted of six or more mice. Two-tailed Student’s *t* test (**a**, **b**) and one-way analysis of variance (ANOVA) with Tukey’s multiple comparison test (**c**) were used for statistical analysis. NS, not significant; *p < 0.05; **p < 0.01; ***p < 0.001; ****p < 0.0001. Values are presented as means ± 1 SD.

It has been shown that antibodies binding with virus surface antigens can cooperate with leukocytes to kill or eliminate virus-infected cells via Fc receptors expressed on the surface of various types of leukocytes^44,45^. To test whether Fcγ receptor (FcγR) is involved in the prevention of RSV infection, we immunized WT and FcγR-null non-obese diabetic (NOD) background mice^46^ by using the immunization protocol already described (Table 1 and Fig. 2a). We confirmed that there was no significant difference in the serum SHe-specific IgG or nasal wash SHe-specific IgA antibody responses between the WT and FcγR-null NOD mice (Fig. 5a, b). At 1 week after the final immunization, mice were challenged intratracheally with 1 × 10^6^ PFU of RSV, and 4 days after the viral challenge, lung viral loads were determined. NI with cCHP-[SHe-PspA]+cdAMP significantly reduced RSV replication in the lungs of WT NOD mice but not in FcγR-null NOD mice (Fig. 5c). These results indicate that SHe-specific serum IgG antibodies induced by NI with cCHP-[SHe-PspA]+cdAMP contribute to the Fc receptor-mediated immune response against lung infection by RSV (e.g., ADCC), just like the SHe-specific IgG antibodies induced by systemic immunization.

### cCHP-[SHe-PspA] does not induce VED

**Fig. 6:**
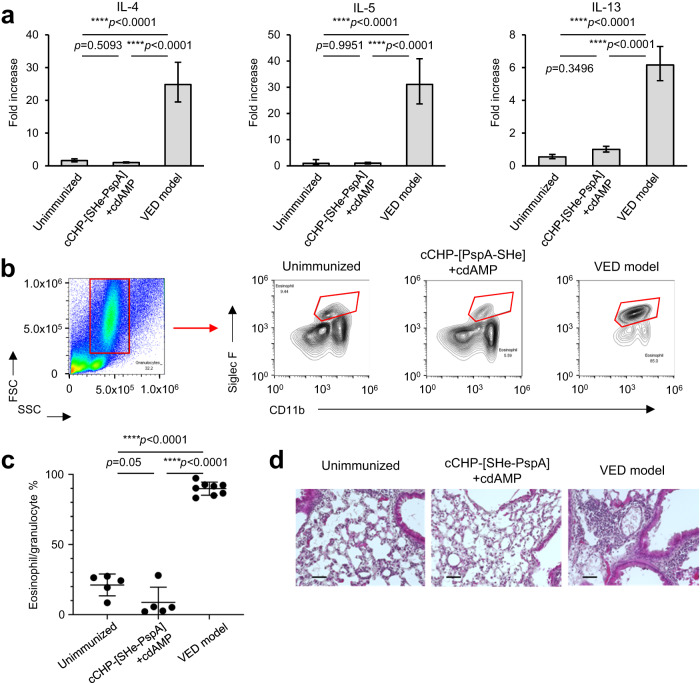
cCHP-[SHe-PspA] did not induce allergic airway inflammation after RSV infection. **a** mRNA expression levels of the Th2-type cytokines interleukin (IL)-4, -5, and -13. **b** Gating strategy for flow cytometric analysis of cells in bronchoalveolar lavage fluid (BALF). Eosinophils were separated as Siglec-F^+^CD11b^+^ cells among the granulocyte population by using forward and side scatter. **c** Eosinophil population among granulocytes in BALF. Data are representative of three independent experiments, and each group consisted of five or more mice. One-way analysis of variance (ANOVA) with Tukey’s multiple comparisons was used for statistical analysis (**a**, **c**). NS, not significant; ****p < 0.001. Values are presented as means ± 1 SD. **d** Sections of lung tissue collected on day 8 after RSV infection were stained with hematoxylin and eosin. Scale bars: 50 µm. Data are representative of three independent experiments, and each group consisted of two or three mice.

VED is a recognized complication elicited by vaccines against RSV^11,12^. We therefore investigated whether NI with cCHP-[SHe-PspA]+cdAMP induced VED by using the FI-RSV VED model previously reported^40^ with slight modifications (Supplementary Fig. 6). VED was evaluated on day 8 after intratracheal RSV challenge in mice NI with PBS or cCHP-[SHe-PspA]+cdAMP by comparing their Th2-type immune response [i.e., production of Th2-type cytokines and eosinophil infiltration into lung tissue^47,48^] with that of FI-RSV VED model mice. The Th2-type cytokine response (i.e., expression of interleukin-4, -5, and -13), as determined by real-time RT-PCR, was comparable between the mice NI with PBS or cCHP-[SHe-PspA]+cdAMP (Fig. 6a). In contrast, significant increases in the cytokine response were observed in the FI-RSV VED model mice (Fig. 6a). Eosinophil infiltration into the BALF was also examined using the Siglec-F^+^ and CD11b^+^ granulocyte fraction, as previously reported^49^ (Fig. 6b), and significantly greater eosinophil infiltration was observed in the FI-RSV VED model mice compared with that in the mice NI with cCHP-[SHe-PspA]+cdAMP (Fig. 6c). Moreover, histological examination of the lungs showed marked eosinophil infiltration and alveolar collapse in the lungs of FI-RSV VED model mice, but no inflammatory cell infiltration or tissue damage in the mice NI with cCHP-[SHe-PspA]+cdAMP (Fig. 6d). These results suggest that cCHP-[SHe-PspA] does not cause VED.

### cCHP-[SHe-PspA] induces SHe-specific immune responses that provide protective effects against RSV in cotton rats

**Fig. 7:**
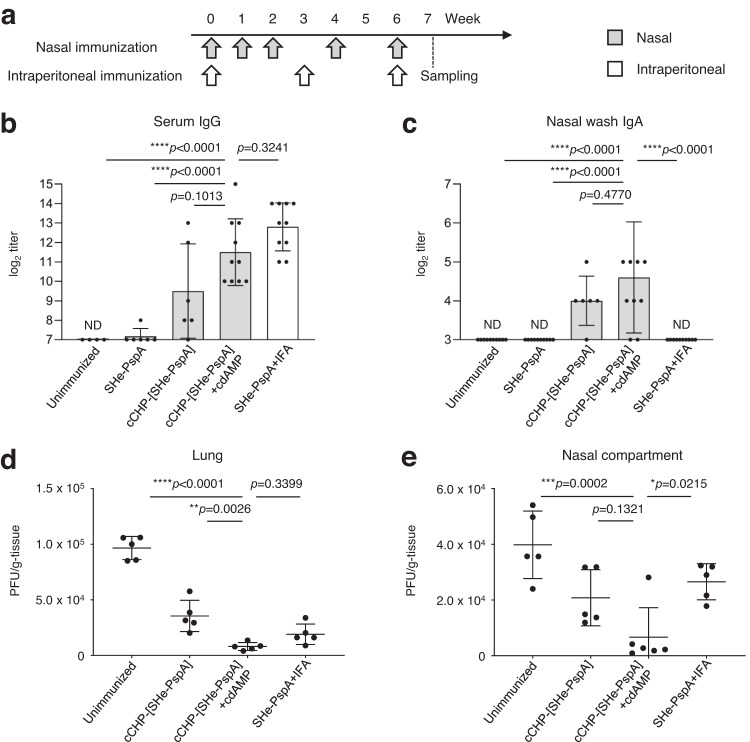
cCHP-[SHe-PspA] induced protective immunity against RSV in cotton rats. **a** Immunization schedule. Wild-type female cotton rats (*Sigmodon hispidus*) were nasally immunized for three times at weekly intervals and then boosted two times at 2-week intervals. Other cotton rats were intraperitoneally immunized every 3 weeks a total of three times. The control group (unimmunized) received PBS administered intranasally. **b**, **c** SHe-specific antibody titers one week after the final immunization in serum and nasal wash. Data are representative of three experiments, and each group consisted of five or more mice. ND, not detected in undiluted samples. **d**, **e** Viral clearance from lungs and nasal compartments, as determined by immunoplaque assay. Data are representative of three independent experiments, and each group consisted of four or more mice (**a**–**d**). One-way analysis of variance (ANOVA) with Tukey’s multiple comparison test was used for statistical analysis. NS, not significant; *p < 0.05; **p < 0.01; ***p < 0.001; ****p < 0.0001. Values are presented as means ± 1 SD.

To further confirm the effectiveness of cCHP-[SHe-PspA], we examined the responses in a standard RSV vaccine efficacy experimental model animal, the cotton rat (*Sigmodon hispidus*)^50–52^. To evaluate whether cCHP-[SHe-PspA] induces protective immunity against RSV infection in both the upper and lower respiratory tracts, cotton rats were NI with PBS, SHe-PspA alone, cCHP-[SHe-PspA], or cCHP-[SHe-PspA]+cdAMP. Other cotton rats were iPI with SHe-PspA+IFA (Table 2 and Fig. 7a). One week after the final immunization, SHe-specific antibody titers in the serum and nasal wash of the cotton rats were determined. Serum SHe-specific IgG titers were elevated in the mice NI with cCHP-[SHe-PspA] or cCHP-[SHe-PspA]+cdAMP or iPI with SHe-PspA+IFA, whereas no SHe-specific IgG was detected in the mice NI with PBS or SHe-PspA alone (Fig. 7b). SHe-specific IgA was induced in the nasal cavity only in the cotton rats NI with cCHP-[SHe-PspA] or cCHP-[SHe-PspA]+cdAMP (Fig. 7a, c. These findings were consistent with the data obtained in mice (Fig. 2b, c).

**Table 2:** Treatment groups used in cotton rat experiments.

Cotton rat treatment groups	SHe-PspA	Adjuvant	Immunization weeks
	/ immunization		
*Unimmunized*			
PBS nasal administration	–	–	–
*Nasal immunization*			
cCHP-[SHe-PspA]	100 µg	–	0, 1, 2, 4, 6
cCHP-[SHe-PspA] + cdAMP	100 µg	cdAMP 5 µg	0, 1, 2, 4, 6
Intraperitoneal immunization			
SHe-PspA + IFA	100 µg	IFA 200 µL	0, 3, 6

Next, we conducted RSV challenge in immunized cotton rats. The cotton rats were intranasally challenged with RSV A2 strain (3.0 × 10^5^ PFU) according to a previously established protocol for evaluating vaccine efficacy against RSV infection in the lungs and nasal compartments^40^. Four days after the viral challenge, viral loads were assessed by immunoplaque assay (Supplemental Fig. 2b). In the cotton rats NI with cCHP-[SHe-PspA]+cdAMP, the viral loads in the lungs were significantly lower than those in the cotton rats NI with PBS or cCHP-[SHe-PspA] (Fig. 7d). The viral load in the unimmunized cotton rats was 9.6 × 10^4^ ± 1.0 × 10^4^ PFU/g. Reductions of viral load were noted in the cotton rats immunized with cCHP-[SHe-PspA] NI, cCHP-[SHe-PspA]+cdAMP NI, or [SHe-PspA]+IFA iPI (3.5 × 10^4^ ± 1.4 × 10^4^, 8.0 × 10^3^ ± 3.6 × 10^3^, and 1.9 × 10^4^ ± 9.2 × 10^3^ PFU/g, respectively). Moreover, cotton rats iPI with SHe-PspA+IFA also showed a low viral load in the lungs (Fig. 7d). In the nasal compartments, NI with cCHP-[SHe-PspA]+cdAMP resulted in significantly reduced viral loads compared with those after NI with PBS or iPI with SHe-PspA+IFA (Fig. 7e). The viral load in the unimmunized cotton rats was 4.0 × 10^4^ ± 1.2 × 10^4^ PFU/g. Reductions of viral load were noted in the cotton rats immunized with cCHP-[SHe-PspA] NI, cCHP-[SHe-PspA]+cdAMP NI, or [SHe-PspA]+IFA iPI (2.1 × 10^4^ ± 1.0 × 10^4^, 6.7 × 10^3^ ± 1.1 × 10^4^, and 2.7 × 10^4^ ± 6.5 × 10^3^ PFU/g, respectively). Together, these findings further indicate that cCHP-[SHe-PspA] is an attractive candidate vaccine for the prevention of RSV infection.

## Discussion

RSV is characterized by its three transmembrane surface proteins: proteins F, G, and SH. The majority of anti-RSV vaccines developed to date use surface protein F as the antigen;^53,54^ and the receptor has been recently identified^55^. In addition, F protein-based vaccines for maternal immunity^20^ and older adults^21,22^ have shown promising results in phase III clinical trials. However, these vaccines have been administered by injection. Moreover, although surface protein F is highly conserved across the spectrum of RSV strains and is essential for viral viability^14^, the RSV virus can make a conformational change to the F protein to avoid antibody neutralization^28,56–59^. Meanwhile, surface protein G is the most variable of the RSV surface proteins^60^, and it induces Th2-biased aberrant immune responses that cause FI-RSV VED via growth arrest-specific 6 signaling^40^. Thus, several obstacles remain to be overcome before an effective nasal vaccine based on these two antigens can be developed. In this study, we have concentrated on developing nasal vaccines using the ectodomain of SH protein (SHe) as the vaccine antigen^30,32^ deployed via our cCHP-nanogel delivery system^23–26,35–38,61^.

SH protein is a 64 (RSV/A) or 65 (RSV/B)-amino acid type II integral membrane protein^62^. Although the function of SH protein is still unclear^63^, it may play a role in viral fusion^64,65^ or in changing membrane permeability^66^. Nevertheless, RSV lacking the SH gene (RSVΔSH) is viable and can replicate just as well as WT RSV^67,68^, indicating that the SH protein is not required for virus entry into host cells^65^. Given this background, SH protein is considered not to elicit high levels of neutralizing antibodies or contribute to host defense upon infection, unlike the other two major surface proteins, F and G^29^. Similarly, SHe, the ectodomain of SH protein, consists of 23 (RSV/A) or 24 (RSV/B) amino acids and appears to have weak immunogenicity^31,33,34^. Indeed, it was reported that SHe-specific IgG levels are very low in convalescent sera from RSV-infected BALB/c mice and cotton rats and in human reference sera^30^. Due to its low immunogenicity^31,33,34^, SHe has not been prioritized for development as a vaccine compared with proteins F and G. Going against this trend, we have developed a new chimeric antigen in which three SHe are linked in sequence to a carrier protein (PspA) (Fig. 1a–c) and then incorporated into an effective nasal delivery vehicle, our cationic-nanogel cCHP^23–26,35–38,61^ (Fig. 1c).

The nasal route of vaccination is likely the most effective strategy to elicit protective immunity against respiratory infections such as RSV. Indeed, nasal vaccination can induce both antigen-specific mucosal and systemic immune responses^69^, whereas conventional injection-type vaccines are less than effective at inducing mucosal immunity despite the induction of effective antigen-specific immune responses in the systemic compartment^70^. In addition, nasal vaccines have the ability to induce high levels of neutralizing IgA antibodies in airway secretions, which is directly associated with a protective immune response against RSV^71–73^. Nasal vaccines induce mucosal immunity by delivering the vaccine antigen to the antigen-sampling classical and respiratory M cells residing in the single-layer epithelium of the nasal cavity, including that of the nasopharyngeal-associated lymphoid tissue and nasal cavity^74^. However, vaccine containing the antigen alone is often poorly immunogenic. To address this issue, we developed a cCHP-nanogel to be used for the delivery of antigen to the nasal mucosa^38^, and we have demonstrated its efficacy and safety in both mouse^23,26^ and non-human primate models^36,37,75^ against *Streptococcus pneumoniae* infection. We therefore used this cCHP-nanogel delivery system to develop a RSV nasal vaccine that contains our chimeric protein antigen.

Here, we found that our newly developed cCHP-based SHe nasal vaccine induced SHe-specific mucosal IgA and systemic IgG antibody responses in mice (Fig. 2b–e). Furthermore, addition of cdAMP as a nasal adjuvant induced significantly higher antibody titers than those elicited without cdAMP (Fig. 2b–e). Immunization with cCHP-[SHe-PspA] plus adjuvant resulted in comparable levels of SHe-specific IgG titers that were seen in mice systemically immunized with SHe-PspA plus IFA (Fig. 2b, d). Here, it should be noted that mucosal SHe-specific IgA antibody responses were induced only in NI mice (Fig. 2c, e). To assess the functional properties of the SHe-specific antibodies, immunized mice were mucosally challenged with live RSV. Our cCHP-based SHe nasal vaccine reduced viral replication in the nasal compartments and lungs (Fig. 3a–f), demonstrating that the vaccine is effective for the control of RSV infection in both the upper and lower respiratory tracts (Fig. 3a–f). The inhibition of RSV replication in the lungs was similar among the immunized mice regardless of immunization routes (Fig. 3a, b, e). In the nasal compartments, mice NI with cCHP-[SHe-PspA]+cdAMP showed a significantly low level of RSV replication compared with that in the other groups of immunized mice (Fig. 3c, d). These results were supported by immunohistochemical analyses using sections of lung and nasal mucosa (Fig. 3e, f). Taken together, these results show that induction of antigen-specific mucosal IgA and serum IgG antibodies plays a central role in the inhibition of RSV replication in the upper and lower respiratory tracts, respectively.

Previous reports have shown that SHe-specific IgG antibodies do not exhibit a direct neutralizing effect against RSV^30,31^. Indeed, we confirmed that SHe-specific serum IgG and nasal wash IgA antibodies induced by our nasal vaccine had no neutralizing effect against RSV (Supplemental Fig. 5). We therefore considered how the SHe-specific IgA antibodies induced in the nasal compartments eliminate RSV in the upper respiratory tract if they do not possess a direct neutralizing function against RSV. We assessed the binding capacities of SHe-specific IgA and IgG antibodies to RSV in nasal washes collected from immunized mice (Fig. 4a, b). SHe-specific IgA and IgG antibodies from nasal washes of mice NI with cCHP-[SHe-PspA] both bound to RSV (Fig. 4a, b); however, the intensity of the binding by IgG was lower than that of IgA. In addition, IgG detected on the upper respiratory mucosa accumulates there via passive diffusion from the serum^76^, whereas IgA is produced continuously by plasma cells resident in the mucosal compartment^77,78^. Although there may be some additive immunity from SHe-specific IgG in the serum in the nasal washes, the major protective immunity was provided by SHe-specific mucosal IgA antibodies.

To further demonstrate the role of SHe-specific nasal IgA antibodies, an intranasal challenge test using RSV pre-incubated with or without nasal washes from mice nasally immunized with our cCHP-based SHe vaccine was conducted. Nasal washes from nasally immunized pIgR^−/−^ mice, which are incapable of secreting mucosal SHe-specific mucosal IgA antibodies, were used for pre-incubation of RSV. RSV pretreated with nasal washes from the immunized pIgR^−/−^ mice caused infection, whereas RSV pretreated with nasal washes from nasally immunized WT mice did not (Fig. 4e). This suggests that SHe-specific IgA antibodies induced by the nasal vaccine function as an active immune defense mechanism in the upper respiratory tract. Indeed, high amounts of the SHe-specific IgA antibodies in the nasal washes could strongly bind RSV (Fig. 4a, c) leading to a significant reduction of virus replication (Fig. 4e). Based on these findings, it appears that SHe-specific IgA antibodies do not exhibit a classical neutralizing effect of inhibiting RSV replication (Supplemental Fig. 5), although they can bind to RSV to inhibit accessing the virus to epithelial cells of the upper respiratory mucosa (Fig. 4a, e).

The SH protein promotes a protective immune response in animal models of RSV infection via an Fc receptor-mediated ADCC mechanism^30,32^. Therefore, it is important to elucidate whether our cCHP-based vaccine-induced SHe-specific IgG antibodies exhibit the same Fc receptor-mediated protective immunity. We found that FcγR-null mice on the NOD background given nasal cCHP-[SHe-PspA] failed to show any protection against RSV challenge, despite the presence of high levels of SHe-specific serum IgG antibodies (Fig. 5a, c). In contrast, WT NOD mice NI with cCHP-[SHe-PspA] did show protection against intratracheal RSV infection and produced high levels of SHe-specific serum IgG antibodies (Fig. 5a, c). These results indicate that Fc receptor-mediated cell-to-cell interactions (e.g., ADCC)^79^ are most likely the protective mechanism of SHe-specific systemic IgG antibodies induced by our cCHP-based nasal vaccine. Supporting this view, it has been reported that some vaccines against influenza virus^80^ and human immunodeficiency virus infection^81^ use ADCC as the protective mechanism against viral infections; these reports show that these vaccines induce viral suppression without the induction of any neutralizing antibodies^80,81^. Therefore, although our results demonstrate that the SHe-specific antibodies induced by our nasal vaccine have no virus neutralizing effect, it is still possible that our vaccine will be able to control RSV infection in the clinical setting because it can induce SHe-specific IgG antibody and Fc receptor-mediated immune responses (e.g., ADCC) for systemic protection as well as SHe-specific IgA antibodies in the nasal compartments that strongly bind to RSV for the blockade of viral invasion at the mucosal surface. A recent non-human primate study with nasal vaccines with F protein-related antigens have also demonstrated similar findings to our SHe based nasal vaccine results (Figs. 4, 5) with the control of upper and lower respiratory infections by virus-specific IgA antibody and Fc-mediated effector mechanisms, respectively^82^. Furthermore, our study provided evidence that cCHP-[SHe-PspA] based nasal vaccine is capable of inducing antigen-specific T cells producing granzyme B or IFN-γ (Supplemental Fig. 1) suggesting possible contribution of cell-mediated immunity. In a separate study, our efforts are currently aiming to characterize more detailed RSV-specific T cell immunity including contributions of residential and circulating SHe-specific T effector and memory cells.

Safety is another major concern in vaccine development, especially for RSV vaccines because in the past there have been cases of severe, harmful side effects and FI-RSV VED caused by RSV vaccines^11,12^. We used the FI-RSV VED model^40^ to assess the safety of our cCHP-based SHe nasal vaccine by examining whether severe Th2-mediated allergic reactions were induced by RSV infection in NI mice. No obvious allergic-type clinical symptoms or aberrant Th2-type responses reminiscent of FI-RSV VED were observed in the nasally immunized mice (Fig. 6a–d). These results provide supportive evidence for the safety of our cCHP-based SHe-PspA nasal vaccine.

The RSV cotton rat model is considered a human-applicable system for vaccine testing because the model shows a similar respiratory pathology after RSV infection as that seen in humans^50–52^. Indeed, the translational value of the cotton rat system is well established^50^, and it has become a recognized model for the study of RSV infection^83^. Using cotton rats, we were able to reproduce the results obtained from our mouse experiments (Fig. 7a–e). Cotton rats NI with cCHP-[SHe-PspA]+cdAMP showed increased levels of SHe-specific serum IgG and mucosal IgA antibodies (Fig. 7b, c). In an intranasal RSV challenge study, cotton rats NI with cCHP-[SHe-PspA]+cdAMP showed protective effects in both the upper and lower respiratory tracts (Fig. 7d, e), with significantly suppressed RSV replication in the upper respiratory tract compared with that in systemically immunized cotton rats (Fig. 7e). Thus, by using the cotton rat model, we were able to provide additional supportive evidence for the efficacy of our cCHP-based SHe nasal vaccine against RSV infection.

The current study has provided a solid base of proof of concept for the induction of protective mucosal and systemic immunity against RSV by nasal immunization with new vaccine candidate formulation of cCHP-[SHe-PspA]. Thus, the study has showed that a weak but safe vaccine candidate antigen, SHe, could be used for the development of a vaccine that uses a nasal vaccine delivery system, cCHP and/or adjuvant candidate cdAMP, by using mouse and cotton rat models. However, we realize that the frequency of immunizations we have adopted in this study might be too many to consider for future clinical application. In our next preclinical study, we will optimize this immunization schedule to be more suitable for clinical practice.

In conclusion, we have demonstrated here that our cCHP-based SHe nasal vaccine provides RSV-specific protective immunity in both the mucosal and systemic compartments of mice and cotton rats without causing VED. Therefore, this formulation is a promising candidate for development as a vaccine for the prevention of RSV infection. Further studies, including clinical tests, are warranted.

## Materials and Methods

### Cells and viruses

RSV strain A2 was obtained from American Type Culture Collection (ATCC, Manassas, VA, USA) (ATCC Number VR-1540^TM^, Lot Number: 70021273). RSV was propagated in cultured HEp-2 cells (ATCC), collected, and stored at −80 °C as stocks. The viral titers of the infectious unit in the RSV stocks were quantified in quadruplicate in HEp-2 cells by immunoplaque assay.

### Animals

BALB/c mice were purchased from Japan SLC (Shizuoka, Japan). pIgR^−/−^ mice on the BALB/c background were maintained by passaging in our laboratory^42,43^, NOD mice were purchased from CLEA Japan (Tokyo, Japan). FcγR-null (both FcR common γ-chain [FcRγ] and FcγRIIB-deficient) mice on the NOD background^46^ were obtained from RIKEN BRC (Tsukuba, Japan). All of the mice were female and 7–8 weeks old at the beginning of the study. Female cotton rats (age, 6 weeks) were purchased from ENVIGO (Indianapolis, IN, USA) via Japan SLC (Shizuoka, Japan). All of the rodents were housed with ad libitum food and water on a standard 12-h/12-h light/dark cycle.

### Preparation of recombinant SHe-PspA protein and cCHP-nanogel formulation

We developed a SHe-PspA antigen in which pneumococcal surface protein A (PspA) was used as a carrier protein for the enhancement of the immunogenicity of SHe^84^. The SHe-PspA vaccine antigen comprised three repeats of SHe peptide of RSV A strain (NKLSEYNVFHNKTFELPRARVNT) at the C-terminus and the PspA sequence from *S. pneumoniae* strain Rx1 (pUAB055; amino acids 1–302) (GenBank accession no. M74122) at the N-terminus. The individual SHe sequences were spaced by a flexible GGGGS linker. The SHe-PspA gene was synthesized by Takara Bio Inc. (Otsu, Japan) and, following digestion with the restriction enzymes Nco I and Xho I (Takara Bio Inc.), inserted into the pET-20b (+) vector (Novagen, Inc., Madison, WI) including a C-terminal His-tag. Rosetta2(DE3) pLysS-competent cells (Novagen, Inc.) were transformed with the SHe-PspA-encoding plasmid according to the manufacturer’s protocol. The resultant transformant was inoculated into lysogeny broth containing 100 µg/mL ampicillin and incubated with shaking at 37°C until the OD600 value was 0.5–0.8. After induction for 3.5 h with 0.4 mM isopropyl β-d-1-thiogalactopyranoside (Wako Pure Chemical Industries, Ltd., Osaka, Japan), the cells were harvested by centrifugation at 4620 × *g* for 15 min at 4 °C and then were extracted as a soluble protein in PBS (pH 7.4) containing 40 mM imidazole and protease inhibitor (cOmplete; Roche Diagnostics K.K., Tokyo, Japan). The desired protein was then purified through salting out with ammonium sulfate (80% saturation) followed by dialysis against a sodium phosphate buffer containing 40 mM imidazole by using a seamless cellulose tube (Sanko Junyaku Co., Ltd., Tokyo, Japan). The protein was then purified by means of nickel affinity chromatography (GE Healthcare Bio-Sciences K.K., Tokyo, Japan) followed by gel filtration on a Sephadex G-100 (GE Healthcare Bio-Sciences K.K.) column (2.5 × 100 cm) using PBS. The purity of the SHe-PspA vaccine was more than 95% by SDS-PAGE (Fig. 1a). After SDS-PAGE with CBB stain, the SHe-PspA band with a molecular weight of 50–55 kDa was cut out of the gel and digested overnight at 37 °C with 0.2 µg of trypsin (Sequence grade, Promega, Madison, WI), desalted, and then concentrated to a volume of 20 mL. The samples were then injected into a direct nanoflow liquid chromatography system (Dina; KYA Technologies) and sprayed into a linear ion trap Orbitrap mass spectrometer (LTQ Orbitrap; Thermo Fischer Scientific, Suwanee, GA) (Fig. 1b). Analysis of the data using the Mascot search server confirmed the complete amino acid sequences of the SHe-PspA expressed in *E. coli*.

The cCHP-nanogel was synthesized as described previously^85^. For formulation of the SHe-PspA vaccine, we mixed the cCHP-nanogel^37^ at a 1:1 molecular ratio with SHe-PspA before incubating for 1 h at 40 °C to incorporate the SHe-PspA into the cationic-charged nanoparticles (Fig. 1c). Lipopolysaccharide contamination of the cCHP nanogel or recombinant SHe-PspA protein was confirmed to be less than 10 endotoxin units/mg protein by a *Limulus* test (Fujifilm Wako Chemicals, Tokyo, Japan).

### Mouse immunization and sample collection

Mice were NI with PBS, SHe-PspA alone, cCHP-[SHe-PspA], or cCHP-[SHe-PspA]+cdAMP (cdAMP, 10 µg; Yamasa Corporation, Chiba, Japan) once a week for 3 consecutive weeks and then boosted two more times at 2-week intervals (10 μg of SHe-PspA protein per immunization). Other mice were iPI with SHe-PspA plus incomplete Freund’s adjuvant (IFA, 100 µL; BD Difco, Franklin Lakes, NJ, USA) or SHe-PspA alone three times once every 3 weeks (Table 1 and Fig. 2a). Serum, nasal wash, and BALF were collected 1 week after the final immunization. Blood samples were obtained from submandibular vein of mice^86^. For collecting the nasal wash samples, a total of 100 μL of sterile PBS was flushed through the anterior nasal cavity by pipetting with 4 µL aliquots of PBS. BALF was harvested by instilling 1 mL of sterile PBS through a blunt needle placed in the trachea.

For immunization, mice were anesthetized intraperitoneally with a mixture of medetomidine, midazolam and butorphanol (MMB)^87,88^. Briefly, medetomidine hydrochloride (Domitor®, ZENOAQ, Koriyama, Japan), midazolam (Dormicum®, Astellas Pharma Inc., Tokyo, Japan) and butorphanol (Vetorphale®, Meiji Seika Pharma Co., Ltd., Tokyo, Japan) were used as MMB anesthetic agents. Medetomidine, midazolam and butorphanol were mixed and diluted with saline (Otsuka Normal Saline®, Otsuka Pharmaceutical Factory, Inc., Tokushima, Japan) to concentrations of 0.06, 0.8 and 1.0 mg/mL, respectively. Then, MMB was administered at the dosing volume of 5 mL/kg.

### Cotton rat immunization and sample collection

Cotton rats were NI with PBS, cCHP-[SHe-PspA], or cCHP-[SHe-PspA]+cdAMP (5 µg; Ajinomoto, Tokyo, Japan) once a week for 3 consecutive weeks and then boosted two more times at 2-week intervals (100 μg of SHe-PspA protein per immunization). Other groups of cotton rats were iPI with SHe-PspA plus IFA (200 µL; BD Difco) three times once every 3 weeks (Table 2 and Fig. 7a). Serum and nasal wash were collected 1 week after the final immunization. Blood samples were obtained from lateral tail vein of cotton rats. For collecting the nasal wash samples, a total 100 μL of sterile PBS was flushed through the anterior nasal cavity by pipetting with 10 µL aliquots of PBS. For immunization, cotton rats were euthanized with 3–4% concentration of isoflurane (Muromachi Kikai Co., Ltd.).

### RSV infection of immunized mice

One week after the final immunization, mice were challenged intratracheally with 1 × 10^6^ PFU of RSV suspended in 50 µL PBS to evaluate vaccine efficacy in the lower respiratory tract, and other mice were challenged intranasally with 1 × 10^5^ PFU of RSV suspended in 12 µL of PBS to evaluate vaccine efficacy in the upper respiratory tract under anesthesia with MMB described as above. Four days after infection, the mice were euthanized, and the lungs and nasal compartments were collected and evaluated for their viral load (Supplemental Fig. 2a).

### Intranasal challenge with RSV pre-incubated with nasal wash from immunized mice

One week after the final immunization, nasal washes were collected from immunized mice. Samples of the nasal wash (7 µL/sample) were diluted in serum-free Dulbecco’s modified Eagle’s medium (DMEM) and then incubated with 1.0 × 10^5^ PFU of RSV for 30 min at 37 °C in a final nasal challenge volume of 12 µL per mouse. After incubation, RSV was administered intranasally to naïve BALB/c mice as described above (Supplemental Fig. 4).

### RSV infection of immunized cotton rats

One week after the final immunization, cotton rats were challenged intranasally with 3 × 10^5^ PFU of RSV suspended in 100 µL PBS under anesthesia with 3–4% concentration of isoflurane described as above to evaluate vaccine efficacy in the upper and lower respiratory tracts. Four days after the infection, the cotton rats were euthanized, and the lungs and nasal compartments were collected and evaluated for their viral load (Supplemental Fig. 2b).

### Antibody titers

Antibody titers of anti-SHe IgG or IgA from immunized mice and cotton rats were determined by enzyme-linked immunosorbent assay^23^. Serum, nasal wash, and BALF samples were prepared in two-fold serial dilutions and loaded into a 96-micro-well plate (NUNC MAXISORP IMMUNO; Thermo Fisher Scientific) coated with 1 μg/mL recombinant SHe conjugated with bovine serum albumin (BSA). Synthesis of the SHe peptides and BSA-SHe for this assay was conducted on our behalf by Eurofins Genomics K.K. (Tokyo, Japan). SHe peptides were synthesized using fluorenyl-methoxy-carbonyl (Fmoc) solid-phase peptide synthesis^89^ and then conjugated chemically with BSA. The target peptide sequence was NH2-C+NKLSEYNVFHNKTFELPRARVNT-COOH. Horseradish peroxidase-conjugated goat anti-mouse IgG (1:4000; #1030-05, SouthernBiotech, Birmingham, AL, USA), goat anti-mouse IgA (1:4000; #1040-05, SouthernBiotech), goat anti-rat IgG (1:4000; #3030-05, SouthernBiotech), and goat anti-rat IgA (1:40,000; #97185, Abcam, Cambridge, UK) were used as the secondary antibodies. Reactions were visualized by using the TMB Microwell Peroxidase Substrate System (XPL, Gaithersburg, MD, USA). The endpoint titer was expressed as the reciprocal log2 of the last dilution that gave an OD_450_ that was 0.1 unit greater than that of the negative control.

### Immunoplaque assay

For the immunoplaque assay, total lungs and nasal compartments were homogenized, and serial dilutions of the supernatant were incubated in a 24-well plate (Corning, NY, USA) with HEp-2 cell monolayers in DMEM and overlaid with 3.0% methylcellulose, as described previously^40,90^. After 3 days, viral plaques were visualized using a goat anti-RSV polyclonal antibody (1:200; #AB1128, Millipore, Billerica, MA, USA) and horseradish peroxidase-conjugated rabbit anti-goat IgG (H+L) secondary antibody (1:100; #61-1620, Thermo Fisher Scientific)^40^. The final visualized plaques were counted and the number of plaques per gram of tissue (PFU/g-tissue) was calculated.

### Plaque reduction assay

The virus neutralization activity of mouse serum and nasal wash was tested by plaque reduction assay using palivizumab (Synagis; AbbVie Inc. North Chicago, IL, USA), an anti-RSV monoclonal antibody, as a positive control. Serum or nasal wash from immunized mice were diluted in serum-free DMEM and incubated with approximately 400 PFU or 25 PFU of RSV, respectively, for 30 min at 37 °C in a final volume of 1.5 mL. These samples were then used to infect a confluent cell layer of HEp-2 cells grown in a 24-well plate (Corning) in DMEM and overlaid with 3.0% methylcellulose^40^. On day 3, the plaque reduction assay was carried out with the anti-RSV antibody, as described above.

### Immunohistochemical analysis for RSV invasion

Lung and nasal cavity samples for confocal microscopy were prepared as described previously^91^. Briefly, the samples were fixed in 4% (wt/vol) paraformaldehyde in PBS overnight at 4 °C with rocking, followed by soaking in 20% (wt/vol) sucrose in PBS overnight at 4 °C with rocking. The samples were then embedded in Super Cryoembedding Medium (Leica Microsystems K.K., Wetzlar, Germany). For immunofluorescence staining, 10-μm-thick frozen sections were prepared by using a CryoJane Tape-Transfer System (Instrumedics, St. Louis, MO, USA) and allowing the sections to air dry. The RSV in the sections was detected by using a goat anti-RSV polyclonal antibody (1:480; #AB1128, Millipore, Billerica, MA, USA) and a donkey anti-goat IgG (H+L) (1:400; #705-166-147, Jackson ImmunoResearch, West Grove, PA, USA). Nasal cavity sections were further stained for actin filament with Phalloidin-iFluro647 Reagent (1:1000; #176759, Abcam). After washing, the specimens were mounted in ProLong Glass Antifade Mountant (Thermo Fisher Scientific) with DAPI (Vector Laboratories, Burlingame, CA, USA) and analyzed by using a DM-IRE2 confocal laser-scanning microscope (Leica Microsystems K.K.).

### Quantitative PCR analysis in mice

Total RNA was isolated from whole lung or nasal compartment using Trizol reagent (Thermo Fisher Scientific). After purification, the RNA was treated with DNase, and 0.5 μg of RNA was reverse transcribed into cDNA using PrimeScript RT master Mix (Takara Bio Inc.). Quantitative PCR was performed in triplicate using SYBR green PCR master mix (Thermo Fisher Scientific) and a StepOne Real-Time PCR System (Thermo Fisher Scientific). The specific primers (Hokkaido System Science, Co., Ltd., Sapporo, Japan) used for the real-time reverse-transcription polymerase chain reaction (RT-PCR) were as follows: *Rsv-f* (forward [F], 5′-aatgatatgcctataacaaatgatcagaa-3′; reverse [R], 5′-tggacatgatagagtaactttgctgtct-3′); *Rsv-g* (F, 5′-ccaaacaaacccaataatgattt-3′; R, 5′-gcccagcaggttggattgt-3′); *Il4* (F, 5′-aagaacaccacagagagtgagctc-3′; R, 5′-tttcagtgatgtggacttggactc-3′); *Il5* (F, 5′-ctctgttgacaagcaatgagacg-3′; R, 5′-tcttcagtatgtctagcccctg-3′); *Il13* (F, 5′-cctggctcttgcttgcctt-3′; R, 5′-ggtcttgtgtgatgttgctca-3′); *Gapdh* (F, 5′-tgacctcaactacatggtctaca-3′; R, 5′-cttcccattctcggccttg-3′); *Actb* (F, 5′-ggctgtattcccctccatcg-3′; R, 5′-ccagttggtaacaatgccatgt-3′). *Gapdh* and *Actb* were used as multiple internal control genes for normalization of real-time RT-PCR data.

Fold changes in transcript expression were calculated by comparing the gene expression in the whole lung or nasal compartment samples in each immunization group, with the level in the respective tissue from mice NI with cCHP-[SHe-PspA]+cdAMP assigned a value of 1.

### ELISPOT assay to evaluate antigen-specific T cell induction

One week after the final immunization, lungs, cervical lymph nodes, and spleens were collected from each mouse mashed in complete RPMI medium, and passed through a cell strainer (mesh, 70 µm). After centrifuging at 500 × *g* for 6 min at 4 °C, CD90.2^+^ T cells were purified by using a MACS separation system (CD90.2 MicroBeads, mouse, Miltenyi Biotec, North Rhine-Westphalia, Germany). Then, the numbers of SHe-specific IFNγ or Granzyme B producing T cells were determined by using an enzyme-linked immunospot (ELISPOT) assay. Briefly, 96-micro-well plates (MultiScreen; Merck, Darmstadt, Germany) were coated with 5 μg/mL anti-mouse IFNγ (#551216, Clone R4-6A2, BD Biosciences, NJ, USA) or 5 μg/mL anti-mouse Granzyme B (#AF1865, R&D Systems, MN, USA) overnight at 4 °C. Plates were washed 3 times with PBS and blocked with complete medium for 1 h at 37 °C. Then, the T cells from each sample were cultured on the pre-captured plate together with the SHe-, SHe conjugated with bovine serum albumin- (SHe-BSA-), or SHe-PspA-pulsed antigen-presenting cells of the T cell-depleted splenocyte population (1.0 × 10^5^/well) at 37 °C for 72 h under 5% CO_2_. To obtain T cell-depleted splenocytes, CD90.2^−^ splenocytes from naïve BALB/c mice were purified by using the MACS separation system to separate the T cells from the other splenic populations. Biotin-conjugated rat anti-mouse IFN-γ (2 µg/mL; #554410, Clone XMG1.2, BD Biosciences) or goat anti-mouse Granzyme B (1 µg/mL; #BAF1865, R&D Systems) were used as a secondary antibody. Spots were developed by incubation for 30 min at room temperature with 3-amino-9-ethylcarbazole (Merck) in 0.1 M sodium acetate buffer, pH 5.0, containing 0.05% H_2_O_2_.

### Preparation of FI-RSV and a murine model of FI-RSV VED

FI-RSV was prepared as previously described^92,93^ with slight modification. In brief, RSV A2 was inactivated with formalin (1:4000) for 72 h at 37 °C. Then, the formalin-treated RSV was resuspended in serum-free medium and inactivation of FI-RSV was confirmed by immunoplaque assay in HEp-2 cells.

A model of FI-RSV VED was created as previously described^40^. In brief, BALB/c mice underwent primary sensitization with FI-RSV (10^5^ PFU equivalent/mouse) and Imject Alum (Thermo Fisher Scientific) via subcutaneous injection. Three weeks later, the mice were sensitized with FI-RSV (10^5^ PFU equivalent/mouse) via intranasal administration three times every other day, followed by a final infection with live RSV (10^6^ PFU/mouse) via intratracheal instillation (Supplemental Fig. 6).

### Flow cytometric analysis of BALF eosinophils

BALF samples were obtained from mice immunized with PBS, cCHP-[SHe-PspA]+cdAMP, or FI-RSV VED on day 8 after RSV challenge, and approximately 2 × 10^6^ cells from the harvested BALF cell suspensions were incubated for 10 min with Fc receptor blocking agent (1:50; #553142, BD Biosciences) to prevent nonspecific binding. Next, the harvested BALF cell suspensions were stained with anti-Siglec F (1:160; #63-1702-80, Thermo Fisher Scientific) and anti-CD11b (1:160; #69-0112-80, Thermo Fisher Scientific) antibodies, and then incubated for 30 min in the dark at 4 °C. Data were acquired on an Attune NxT flow cytometer (Thermo Fisher Scientific) and analyzed using the FlowJo software package (TreeStar, Ashland, OR, USA) (Fig. 6b).

### Histological analysis for VED evaluation

Lung samples from individual mice were fixed in 4% (wt/vol) paraformaldehyde in PBS, embedded in paraffin blocks (Fujifilm Wako Chemicals), and sectioned into a thickness of 5 µm. The sections were then stained with hematoxylin and eosin to assess pulmonary histopathologic changes and eosinophil infiltration, respectively, as described previously^94^.

### Antibody binding assay

RSV was conjugated with Dynabeads Intact Virus Enrichment (#10700D, Thermo Fisher Scientific) in accordance with the manufacturer’s instructions in such a way that the RSV was 1.0 × 10^7^ PFU per 1 µL of beads. The RSV-Beads were then incubated with 70 µL of nasal wash from immunized mice for 0.5 h at room temperature, followed by staining with FITC-conjugated anti-mouse IgG (1:100; #115-096-062, Jackson ImmunoResearch) or biotin-conjugated anti-mouse IgA (1:1000; #1040-08, SouthernBiotech) and allophycocyanin–streptavidin (1:1000; # 20-4317-U100, Tonbo Biosciences, San Diego, CA, USA). Antibody binding was assessed by measuring the intensity of fluorescent labeling by flow cytometry, and the mean fluorescent intensity of each group was measured and expressed with the value for RSV-Beads alone set to 1. Flow cytometric analysis was performed by using an Attune NxT flow cytometer, and all data were analyzed using the FlowJo software package (Supplemental Fig. 3).

### Euthanasia of rodents

Only when necessary, mice and cotton rats were euthanized with decapitation, under anesthesia with 5% or greater concentration of isoflurane (Muromachi Kikai Co., Ltd.), for sample collection.

### Statistical analysis

Comparisons among groups were performed by using two-tailed Student’s *t* test (for 2-group analysis), and one-way analysis of variance (ANOVA) with Tukey’s multiple comparison test (for multiple-group analysis). All statistical analyses were performed using the GraphPad Prism software version 9.0 (GraphPad Software, San Diego, CA). *P-*values less than 0.05 were considered significant.

### Study approval

All animal experiments were performed according to the Guidelines for Use and Care of Experimental Animals of the Institute of Medical Science, The University of Tokyo, Japan, and the protocol was approved by the institute’s Animal Care and Use Committee (approval #PH3-33 for mice and #PA21-12 for cotton rats).

### Reporting summary

Further information on research design is available in the Nature Research Reporting Summary linked to this article.

## Supplementary information

Supplementary Information

Figure Data Sets

REPORTING SUMMARY

## Data Availability

The Supplementary Data underlying Figs. 1b, 2b–e, 3a–d, 4a–e, 5a–c, 6a, c and 7b–e and Supplementary Figs 1a–f and 5a, b are provided as a Source Data file.
