# Supplementary material for: Cationic-nanogel nasal vaccine containing the ectodomain of RSV-small hydrophobic protein induces protective immunity in rodents
Source: NPJ Vaccines. 2023 Jul 24;8:106. doi: 10.1038/s41541-023-00700-3 (PMC10366164; doi:10.1038/s41541-023-00700-3)
Supplement: Supplementary file 1 — Supplementary Information [file 41541_2023_700_MOESM1_ESM.pdf]

# Supplementary information for

## Cationic-nanogel nasal vaccine containing the ectodomain of RSV-small hydrophobic protein induces protective immunity in rodents.

**Umemoto S et al**

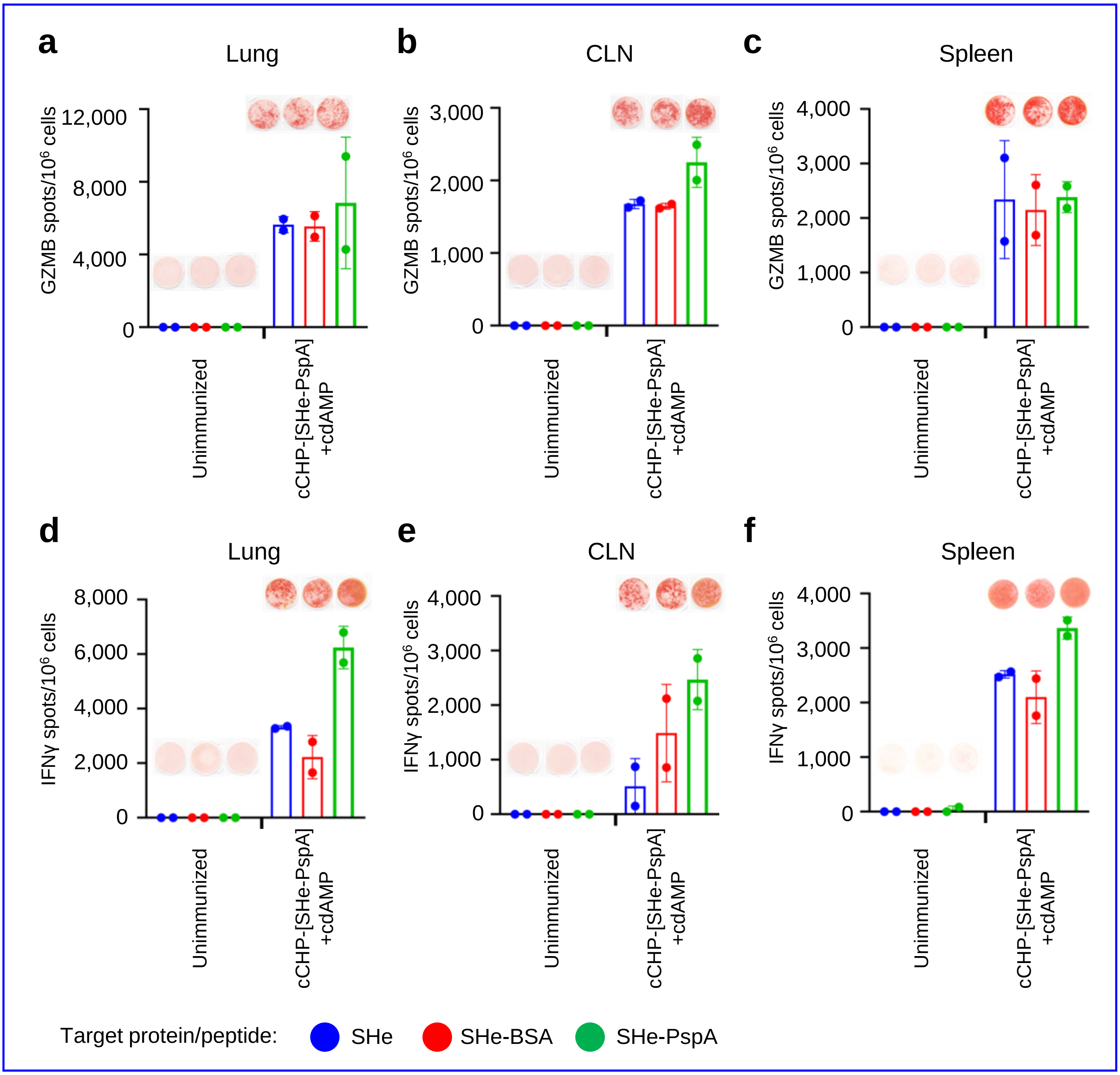


**Supplementary Figure 1. Induction of SHe-specific T cell responses by nasal immunization with cCHP-[SHe-PspA] based vaccine in mice. (a-c)** ELISPOT assay show that SHe-specific T cells producing granzyme B were induced in lungs (a), cervical lymph nodes (CLNs) (b) and spleens (c), after nasal immunization of mice with the cCHP-[SHe-PspA]+cdAMP. **(d-f)** IFNγ-producing SHe-specific T cells were also induced in lungs (d), CLNs (e) and spleens (f) of these nasally-immunized mice. Antigen-specific T cell analyses were conducted by the use of SHe (Blue), SHe-BSA (Red) and SHe-PspA (Green) as detection antigens. Data are representative of two independent experiments. n=2 mice per group; IFNγ, interferon gamma; GZMB, granzyme B. Dots represent ELISPOT results for individual mice.

Umemoto *et al*., Supplementary Figure 1.

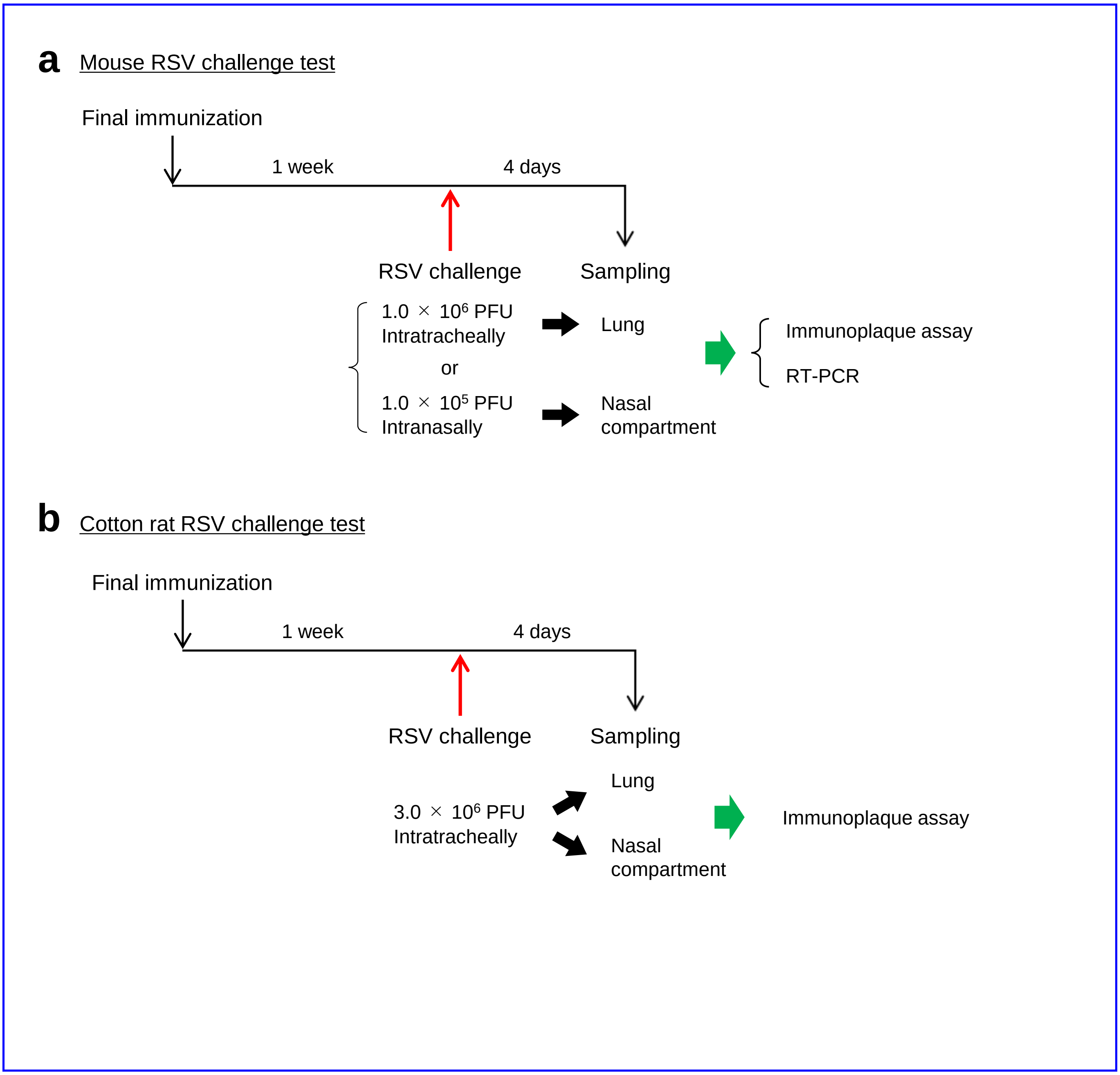


**Supplementary Figure 2. RSV challenge test. (a)** Mouse RSV challenge test . At 1 week after the final immunization, the mice were challenged intratracheally with 1 $\times$ $10^6$ PFU of the RSV A2 strain to evaluate vaccine efficacy in the lungs, or intranasally with 1 $\times$ $10^5$ PFU to evaluate vaccine efficacy in the nasal compartments. At 4 days after the viral challenge, viral titers in the lungs or nasal compartments were determined by immunoplaque assay. Viral loads were determined by detecting RSV-F and -G mRNA using RT-PCR. **(b)** Cotton rat RSV challenge test . At 1 week after the final immunization, the mice were intranasally challenged with 3 $\times$ $10^5$ PFU of the RSV A2 strain to evaluate vaccine efficacy both in the lungs and nasal compartments. At 4 days after the viral challenge, viral titers in the lungs or nasal compartments were determined by immunoplaque assay.

Umemoto *et al*., Supplementary Figure 2.

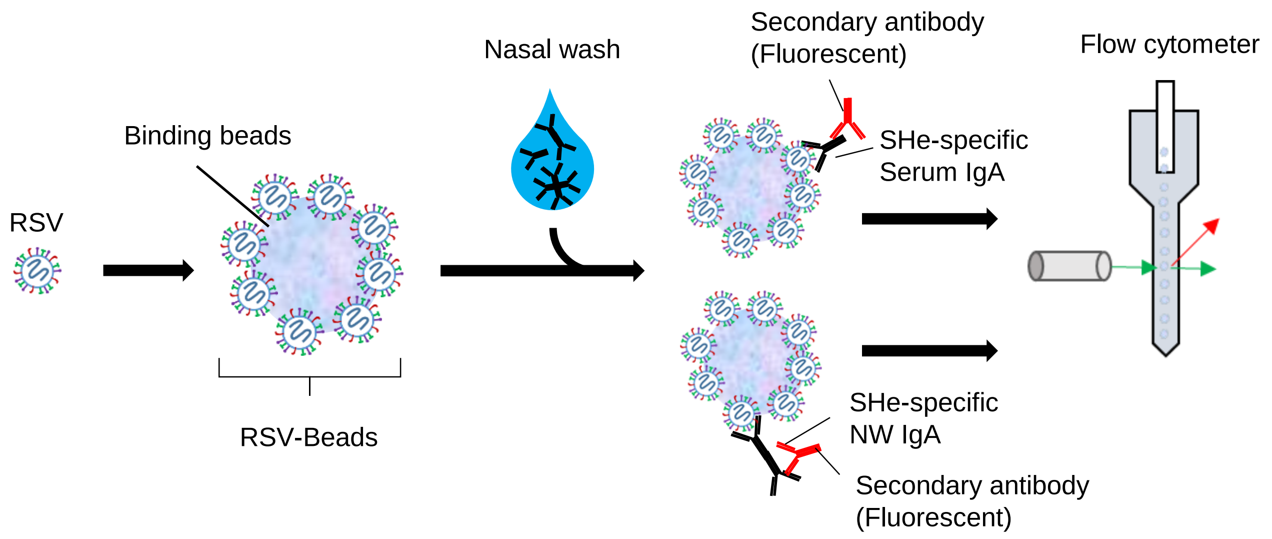


**Supplementary Figure 3. Protocol for the SHe-specific antibody binding assay.** To achieve a particle size that can be detected by flow cytometry, RSV was conjugated with binding beads (RSV-Beads). The RSV-Beads were then incubated with serum or nasal washes obtained from immunized mice. After incubation, the RSV-Beads were reacted with anti-mouse IgG or IgA fluorescent antibodies, and fluorescence was evaluated by fluorescence-activated cell sorting.

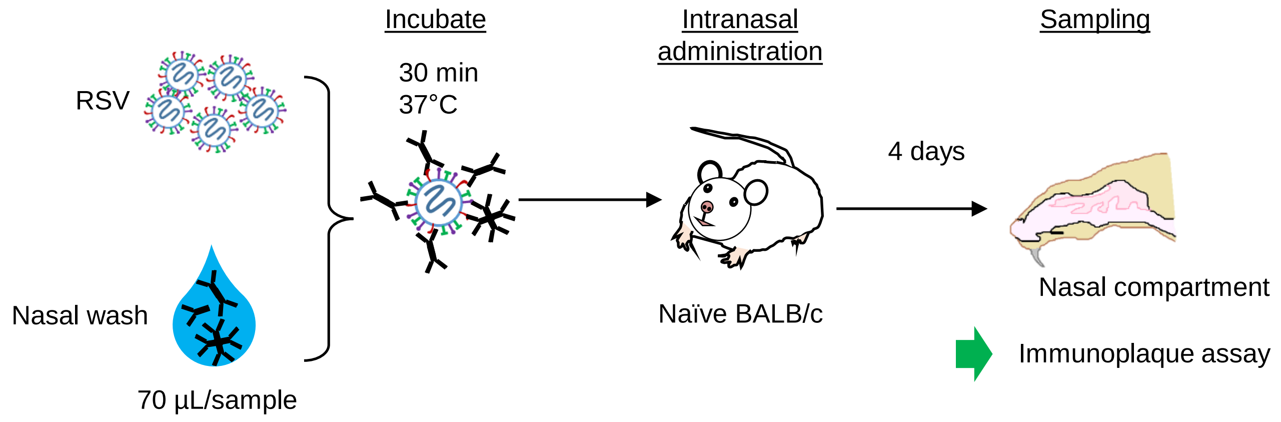


**Supplementary Figure 4. Protocol of the intranasal challenge test of RSV pre-incubated with nasal wash from immunized mice.** RSV A2 was pre-incubated with nasal wash obtained from immunized mice. Then, immunization-naïve BALB/c mice were challenged with the pre-incubated RSV. At 4 days after viral challenge, the viral titer in the nasal compartments of the mice was determined by immunoplaque assay.

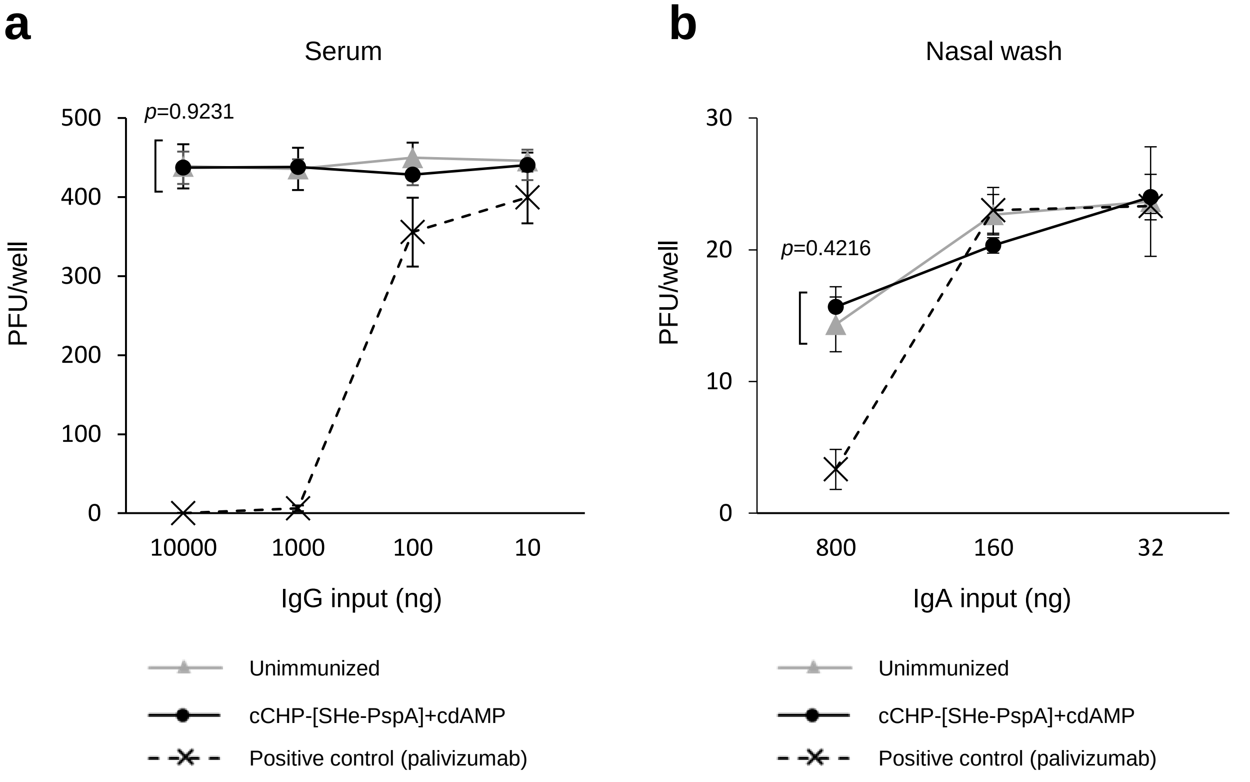


**Supplementary Figure 5. Antibodies induced by cCHP-[SHe-PspA] do not have a direct neutralizing effect against RSV. (a, b)** Serum or nasal wash from mice nasally immunized with cCHP-[SHe-PspA] did not directly neutralize RSV in vitro. An RSV A2 in vitro neutralization assay was conducted by using the indicated dilutions of pooled sera or nasal washes from mice at one week after the final immunization. Viral antigen titer was quantified by plaque reduction assay using palivizumab as a positive control. Two-tailed Student's *t*-test was used for statistical analysis. Values are presented as means ± 1 SD.

Umemoto *et al*., Supplementary Figure 5.

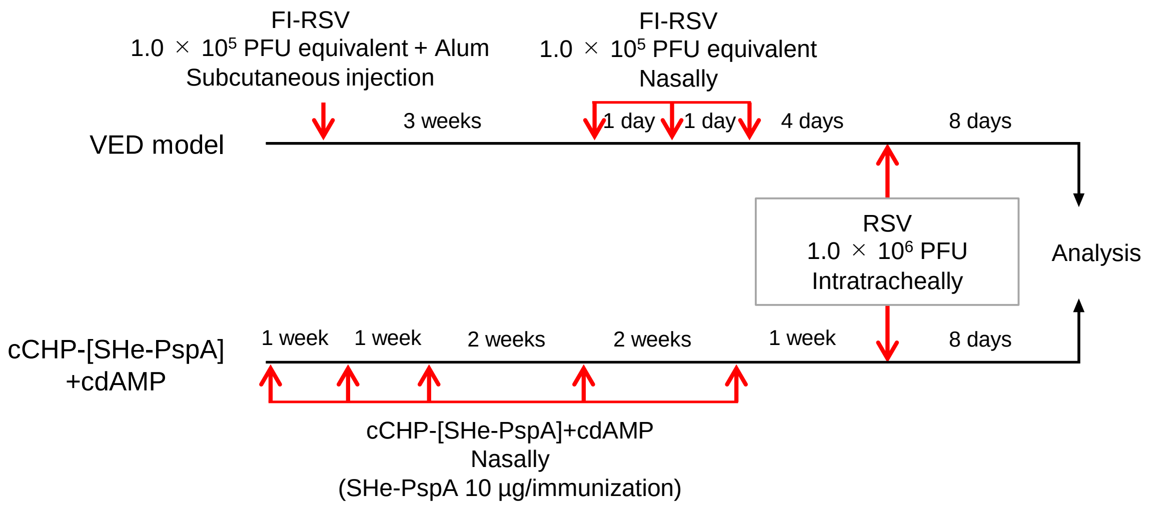


**Supplementary Figure 6. Schedule for evaluation of VED.** Timeline for establishing a model of formalin-inactivated-RSV vaccine-enhanced disease (FI-RSV VED), and the protocol used to compare the model with mice immunized with the cCHP-based SHe nasal vaccine.
